# DNA methylation associates with survival in non-metastatic clear cell renal cell carcinoma

**DOI:** 10.1186/s12885-019-5291-3

**Published:** 2019-01-14

**Authors:** Emma Andersson Evelönn, Mattias Landfors, Zahra Haider, Linda Köhn, Börje Ljungberg, Göran Roos, Sofie Degerman

**Affiliations:** 1 Department of Medical Biosciences, Umeå University, NUS, Blg 6M, 2nd floor, SE-90185 Umeå, Sweden; 2 Department of Radiation Sciences, Umeå University, Umeå, Sweden; 3 Department of Surgical and Perioperative Sciences, Urology and andrology, Umeå University, Umeå, Sweden

**Keywords:** Clear cell renal cell carcinoma, DNA methylation, Prognosis, Genetic

## Abstract

**Background:**

Clear cell renal cell carcinoma (ccRCC) is the most common subtype among renal cancer and is associated with poor prognosis if metastasized. Up to one third of patients with local disease at diagnosis will develop metastasis after nephrectomy, and there is a need for new molecular markers to identify patients with high risk of tumor progression. In the present study, we performed genome-wide promoter DNA methylation analysis at diagnosis to identify DNA methylation profiles associated with risk for progress.

**Method:**

Diagnostic tissue samples from 115 ccRCC patients were analysed by Illumina HumanMethylation450K arrays and methylation status of 155,931 promoter associated CpGs were related to genetic aberrations, gene expression and clinicopathological parameters.

**Results:**

The ccRCC samples separated into two clusters (cluster A/B) based on genome-wide promoter methylation status. The samples in these clusters differed in tumor diameter (*p* < 0.001), TNM stage (*p* < 0.001), morphological grade (*p* < 0.001), and patients outcome (5 year cancer specific survival (pCSS_5yr_) *p* < 0.001 and cumulative incidence of progress (pCIP_5yr_) *p* < 0.001. An integrated genomic and epigenomic analysis in the ccRCCs, revealed significant correlations between the total number of genetic aberrations and total number of hypermethylated CpGs (R = 0.435, *p* < 0.001), and predicted mitotic age (R = 0.407, *p* < 0.001). We identified a promoter methylation classifier (PMC) panel consisting of 172 differently methylated CpGs accompanying progress of disease. Classifying non-metastatic patients using the PMC panel showed that PMC high tumors had a worse prognosis compared with the PMC low tumors (pCIP_5yr_ 38% vs. 8%, *p* = 0.001), which was confirmed in non-metastatic ccRCCs in the publically available TCGA-KIRC dataset (pCIP_5yr_ 39% vs. 16%, *p* < 0.001).

**Conclusion:**

DNA methylation analysis at diagnosis in ccRCC has the potential to improve outcome-prediction in non-metastatic patients at diagnosis.

**Electronic supplementary material:**

The online version of this article (10.1186/s12885-019-5291-3) contains supplementary material, which is available to authorized users.

## Background

Clear cell renal cell carcinoma (ccRCC) is the most common histological subtype accounting for 80–90% of all RCCs. Clear cell RCC is associated with few clinical symptoms; i.e. flank pain, hematuria or a palpable abdominal mass, but is nowadays mostly discovered incidentally due to extensive use of computed tomography (CT), ultrasound, and magnetic resonance tomography (MRT) [1]. This has led to earlier discovery of tumors, and the number of patients with metastases at diagnosis has decreased to 18% in Sweden [2]. If metastases are present at diagnosis, the probability of 5-year survival (pOS_5yr_) may be as low as 10–15% [3]. Among patients with local disease, the prognosis is better (pOS_5yr_ up to 90% with modern protocols), but still 20–30% of patients with local disease at diagnosis will develop metastasis after nephrectomy [3]. In addition to TNM stage and morphological grade [4, 5], there is a need for new molecular markers to identify patients with high risk of progress.

Genetic alterations in ccRCC show considerable heterogeneity. Loss of chromosome 3p and inactivation of the *VHL* (von Hippel–Lindau) gene are frequently observed [6]. Gain of chromosomes 1q, 3q, 7q, 8q, 20q; loss of chromosomes 1p, 4p, 4q, 9p, 9q, 13q, 14q and loss of whole chromosomes 4, 9, 19, 20 and 22, have all been reported in ccRCC [7–12].

Several studies have aimed at identifying molecular markers that predict survival in ccRCC. Gene expression alterations have been associated with prognosis [13–26], but none of these genes are currently clinically used.

DNA methylation has emerged as an important regulator of gene expression, and has been implicated in both cancer development and progression. DNA methylation on Cytosine-phosphate-Guanine (CpG) sites in promoter regions may alter the affinity of transcription factors for their binding sites, and may also, in combination with chromatin modifications, contribute to silencing of genomic regions [27]. Altered DNA methylation has been identified as a prognostic marker, as well as a potential target for therapy, in several malignancies [27, 28]. De novo methylated CpGs in ccRCC assumed to be of relevance for RCC tumorigenesis have been identified, but their clinical value requires further validation [29, 30]. Arai et al., (2012) and Tian et al., (2014) identified CpG island methylator phenotype (CIMP) panels using the Infinium HumanMethylation27K array and MassARRAY, respectively, that predicted cancer-free survival and overall survival [31, 32]. In 2015, Wei et al. presented a CpG-methylation-based assay using the Illumina HumanMethylation450K array, calculating a risk score that predicted overall survival independently of clinicopathological parameters in ccRCC [33].

We have previously shown that genome-wide promotor methylation status can predict survival in ccRCC [34]. Using Illumina HumanMethylation27K arrays, we found a stepwise increase in methylation with TNM stage and morphological grade. In the present study, we increased the number of patients and performed a detailed analysis of promoter associated CpGs by Illumina HumanMethylation450K arrays. Thereby, we further investigated the prognostic value of alterations associated with tumor progression. Identifying methylation patterns at diagnosis unique for non-metastatic patients with high risk of later progress is important since these patients may need adjuvant treatment and/or more frequent follow up to improve survival.

## Methods

The aim with this study was to evaluate the prognostic relevance of DNA methylation in relation to clinical characteristics in ccRCC, with special focus on non-metastatic patients at diagnosis.

### Patients and tissue samples

The study cohort consisted of 115 ccRCC patients, primary treated with radical or partial nephrectomy between 2001 and 2009, and diagnosed at the University hospital in Umeå, Sweden. None of the patients received neoadjuvant or adjuvant therapy. Eighty-seven patients were metastasis-free (M0), while 28 had metastases (M1) at diagnosis. Tumor free (TF) tissue samples were obtained from 12 surgically removed tumor bearing kidneys and were considered histologically normal by a pathologist. The tumor and TF tissue samples obtained were snap-frozen in liquid nitrogen, and stored in − 80 °C until analysis.

Patients were followed-up at least yearly by routine clinical and radiological examination in accordance with a scheduled follow-up program. Clinical follow-up data were extracted in August 2017. All patients have given informed consent and the study was approved by the regional ethical review board in Umeå (Dnr 2011–156-31 M, 20110523).

The publically available TCGA-KIRC dataset was used as a validation cohort and clinical information was downloaded from the Broad Institute’s Genome Data Analysis Center Firehose (http://gdac.broadinstitute.org/). Only unique non-metastatic (M0) ccRCC samples (technical replicates excluded) analysed with Illumina HumanMethylation450K array were included in the analysis (*n* = 230). All patients were treated with radical or partial nephrectomy and patients receiving neoadjuvant and/or adjuvant therapy were excluded.

### Methylation array analysis

DNA was extracted from the tissue samples as described previously [35] and was subjected to bisulfite conversion (500 ng of each sample) using the EZ DNA Methylation Kit (Zymo Research, Irvine, USA) according to the manufacturer’s protocol. Bisulfite DNA conversion was verified by MethyLight analysis of the *ALU* gene with the ALU-C4 primer/probe set as described [36]. Genome-wide assessment of DNA methylation was performed using HumanMethylation450K BeadChip arrays (Illumina, San Diego, CA, USA) according to manufacturer’s protocol. To each array, 200 ng of bisulfite-converted DNA was applied, and the arrays were scanned with a HiScan array reader (Illumina). The fluorescence intensities were extracted using the Methylation module (1.9.0) in the Genome Studio software (V2011.1). Pre-filtering and normalization steps are shown in Additional file 1: Table S1 and were performed as previously described [37, 38], which excluded the X and Y chromosomes, CpGs with detection *p*-value > 0.05 and CpG probes that aligned to multiple loci in the genome or were located less than 3 bp from a known single nucleotide polymorphism [39]. The methylation levels (i.e., the β value) of each CpG sites ranges from 0, corresponding to completely unmethylated DNA, to 1, representing fully methylated DNA. The technical reproducibility of methylation array analysis was monitored by including a replicate sample on each array and the R^2^-values ranged from 0.995 to 0.997.

The analysis was restricted to the *n* = 155,931 CpGs located in gene promoter regions, i.e. located within TSS1500, TSS200 and 5’UTR, remaining after the initial filtration steps (Additional file 1: Table S1). Twelve TF tissue samples were included as reference samples. The TF-samples showed a high similarity in promoter methylation, the R^2^-values ranged from 0.96 to 0.99. A CpG site was determined as differently methylated (DM-CpG) if the absolute value of the difference in beta value between tumor sample and the mean of TF samples (Δβ) was greater than or equal to 0.2. The DM-CpGs in the M0-PF (non-metastatic progression free), M0-P (non-metastatic with later progress) and M1 (metastasis at diagnosis) groups were analysed against known methylation quantitative trait loci (mQTLs) [40] based on middle-aged individuals to evaluate genetic variants versus cancer specific alterations, and these sites were excluded from further analysis.

The epigenetic mitotic clock described by Yang et al., 2016 was used to estimate mitotic age [41]. A prognostic Risk score was calculated for 114 out of 115 tumor samples using the CpG-methylation-based assay previously presented by Wei et al., (2015) [33]. Patients with a Risk score higher than − 0.1 were defined as high risk and lower than − 0.1 were defined as low risk, as previously stated [33].

DM-CpGs within the M0-PF, M0-P and M1 groups were selected for further analysis if differently methylated in at least 70% of samples (in Fig. 7). Hypermethylated CpGs showed increased methylation compared to TF whereas hypomethylated CpGs were less methylated.

The commonly DM-CpGs (*n* = 172) in the M0-P and M1 samples were defined as a Promoter Methylation Classification (PMC) panel. The hypomethylated CpGs (*n* = 51) were mirrored (1 – average beta) and thereafter an average beta of the 172 DM-CpGs were calculated for each sample. A ROC-curve was constructed with PMC average beta as test variable and tumor progression within five years as state variable. Youden index was used to determine the cut off for PMC groups (PMC high/low), and was set to PMC average beta 0.688 (specificity 0.85 and sensitivity 0.55).

The prognostic relevance of PMC classification was confirmed in a separate ccRCC cohort (*n* = 230) within the KIRC project of TCGA [42]. Clinical information along with methylation raw data (Illumina HumMeth450Karrays) were downloaded from the Broad Institute’s Genome Data Analysis Center Firehose (http://gdac.broadinstitute.org/). Beta values were constructed using the genome studio definition and was normalized for different bead types using BMIQ. Missing values among the 172 CpG-sites in the PMC panel were imputed using the k-nearest neighbours method. Deaths without prior progression were counted as non-events and were censored.

The distribution of hyper- and hypomethylated CpGs within twelve genomic regions with frequent gain/loss in ccRCC (as defined by Köhn et al. 2014 [9] and listed in Additional file 2: Table S3) was analysed to identify potential overrepresentation of hyper- and/or hypomethylated CpGs within these regions.

### Heterogeneity analysis

In six individuals, multiple tumor samples were taken from different locations within the same kidney to study intratumoral heterogeneity. To confirm that the collected samples originated from the same individual, the methylation levels of the 65 built in SNP probes (in the methylation array) were analysed. One of the 6 patients (number 3) was excluded at this step due to signs of contaminated DNA (Additional file 3: Figure S1).

### Genomic aberrations

The genetic aberrations were profiled using the total intensity signals of the raw data from the HumanMethylation450K arrays [43, 44]. Briefly, copy number variation (CNV) analysis was performed in R (v3.4.1) using the Conumee package (v1.9.0) [45] with data imported through minfi (v1.18) [46]. Parameters and limits for calling deletion and gain were set for each sample individually through visual inspection.

The validity of this method to identify CNV was confirmed by comparing the methylation results with genetic aberrations identified by HumanCytoSNP-12 v2.1 arrays (Illumina) in a subset of samples (*n* = 57 ccRCC) [9]. The CNV analysis was performed in Genome Studio v1.8 using the Genotyping Module (Illumina). Cohen’s kappa test was used to compare the results from the two array types, which were significantly overlapping (*p* ≤ 0.001 for all analysed aberrations), with quality of agreement moderate or good (Additional file 4: Table S2).

The genomic aberrations for all ccRCCs were summarized by investigating the minimal overlapping regions of commonly occurring CNVs in ccRCC [9]. The CNVs are presented as percentage of samples where alterations across the entire regions were found (Additional file 5: Figure S2 and Additional file 2: Table S3).

### RNA preparation and gene expression array analysis

RNA was extracted from 28 tumors using MagAttract RNA Universal Tissue M48 Kit (Qiagen, Hilden, Germany) according to manufacturer’s protocol using BioRobot M48. RNA concentrations were determined by spectrophotometry (NanoDrop, Thermo Scientific, Wilmingron, DE, USA) and quality was analysed using the 2100 Bioanalyzer (Agilent technologies, Santa Clara, CA, USA).

Two hundred ng of total RNA from each sample was used for cRNA production by the Illumina TotalPrep RNA amplification kit (Ambion Inc., St. Austin, TX, USA) according to the provided protocol. The quality of purified cRNA was evaluated using the RNA 6000 p kit (Agilent Technologies) in the Agilent 2100 Bioanalyzer (Agilent Technologies). A total of 750 ng biotinylated cRNA was hybridized to the human HT12 Illumina Beadchip gene expression array (Illumina, San Diego, CA, USA) according to manufacturer’s protocol and scanned using the Illumina Bead Array Reader (Illumina). Expression array data was analysed using the Illumina BeadStudio V2011.1 software and samples were normalized using the cubic spline algorithm. Gene expression levels of the *MX2* (ILMN_2231928), *SMAD6* (ILMN_1767068) and *SOCS3* (ILMN_1781001) genes were extracted from the arrays.

### Statistical and bioinformatical analysis

For statistical analysis, the Statistical Package for the Social Sciences (SPSS Inc., Chicago, IL) software version 24, was used. The chi-square/Fisher’s exact test was used to compare differences between subgroups among categorical variables and the Mann–Whitney U test was used for continuous variables. Kruskal Wallis test was used for continuous variables when comparing three groups of samples. Mann–Whitney U test with Bonferroni correction was used in Additional file 6: Figure S4.

Estimates of 5-year cancer specific survival (pCSS_5yr_) rates and Cumulative incidence of Progress (CIP_5yr_) in subgroups of ccRCC were obtained from Kaplan–Meier survival tables, and the equality of survival distributions for the groups was compared using the log rank test. The significance level used in all tests was 0.05. In CIP analysis local, regional or distant metastatic progression was the endpoint. In the CSS analysis, ccRCC specific death was the endpoint.

Hierarchical clustering was performed using the Ward’s method and a Euclidean distance metric for clustering samples.

Principal Component Analysis (PCA) was performed in SIMCA version 14 (Umetrics, Umeå, Sweden) after centering the average beta values of 155,931 promoter associated CpG sites.

WebGestalt [47] was used to analyse functional enrichment of the genes associated with progress of disease (specified in Fig. 7a), using all genes represented by the 155,931 promoter associated CpGs as the background list. The gene functions of potential relevance for ccRCC pathology were selected among the 20 most significant GO Terms (Additional file 7: Table S8).

## Results

### DNA methylation classification holds prognostic relevance in ccRCC patients

One hundred and fifteen tumor tissue samples from ccRCC patients along with 12 adjacent tumor free (TF) kidney-cortex tissue samples were analysed using the genome-wide HumanMethylation450K arrays.

**Fig. 1:**
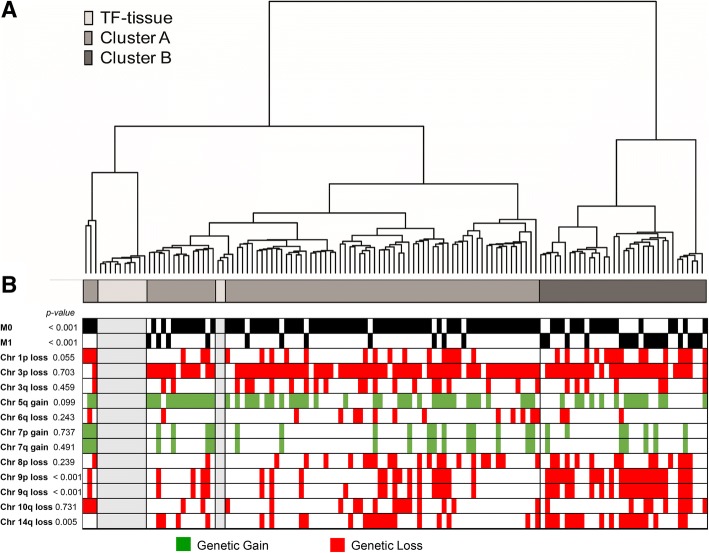
Analysis of promoter associated DNA methylation and genomic aberrations in clear cell renal cell carcinoma (ccRCC) and tumor free (TF) adjacent tissue samples. **a** Cluster analysis, based on 155,931 promoter associated CpGs, on 115 ccRCC and 12 TF-tissue samples divide tumors into two groups, cluster **a** and **b**. **b** Allocation of common genetic aberrations depending on cluster profile. White colour indicates no aberration, red colour genetic loss, and green colour genetic gain. *P*-values compare differences between cluster A/B using Chi-Square test

**Fig. 2:**
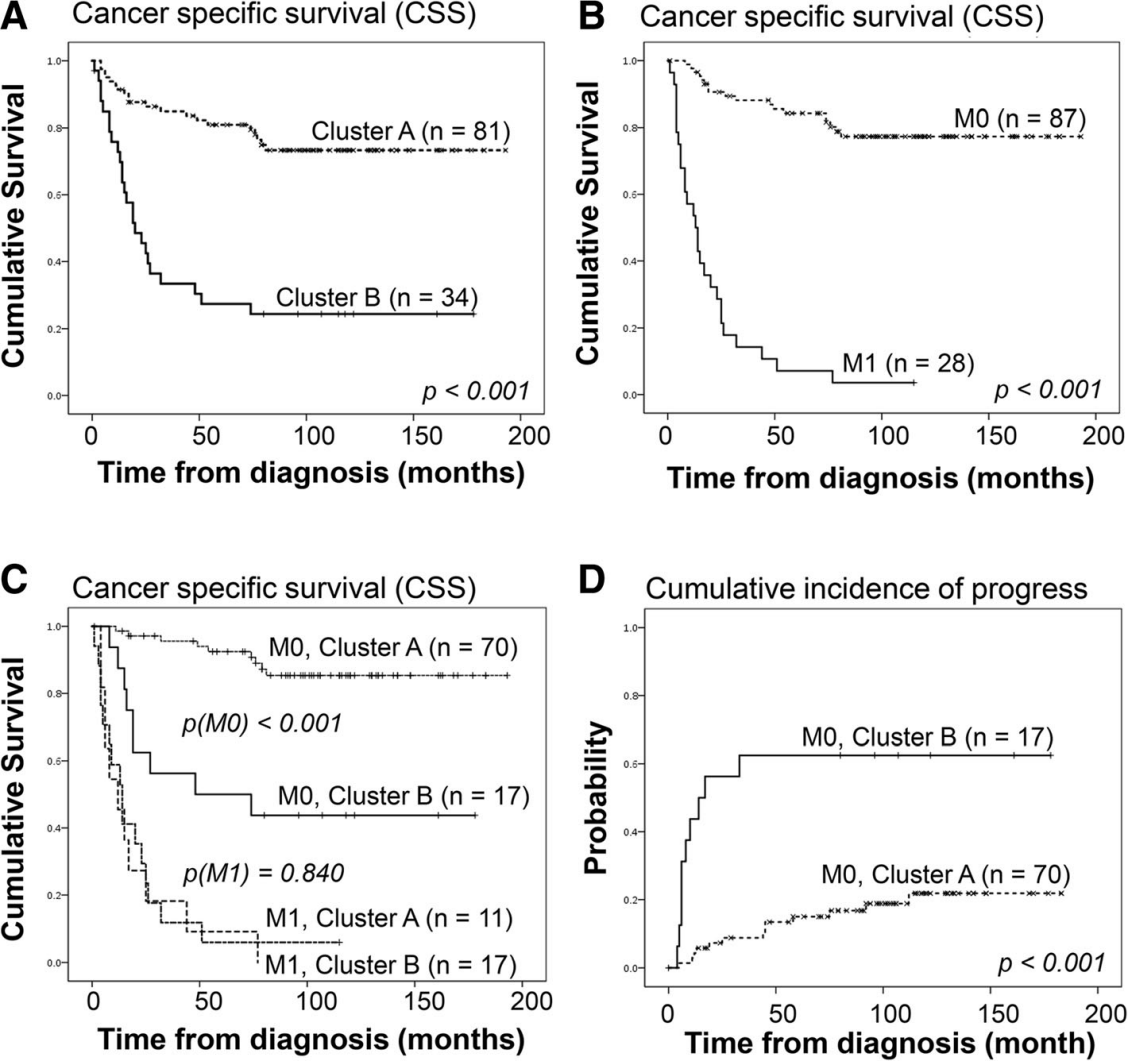
Survival analysis based on M0/M1 and methylation cluster status at diagnosis. Kaplan-Meier cancer specific survival (pCSS_5yr_) analysis in 115 patients with ccRCC, in relation to (**a**) cluster status; (**b**) presence of distant metastasis at diagnosis and (**c**) combination of cluster status and presence of distant metastasis at diagnosis. **d** Cumulative incidence of progress (CIP) analysis in 87 non-metastatic (M0) ccRCC in relation to cluster status. Log-rank *p*-values are presented

We have previously shown that genome-wide promotor methylation status based on Illumina Human Methylation 27 K arrays can predict survival in ccRCC [34]. To further examine the prognostic relevance of promoter methylation status with regard to cancer specific survival and progress free survival we focused the analysis to 155,931 promoter associated CpGs on the HumanMethylation450K array, in metastatic and non-metastatic ccRCCs. Hierarchical clustering of the 155,931 promoter associated CpGs divided the ccRCCs (*n* = 115) and the TF samples (*n* = 12), into two clusters; A (*n* = 81 tumor tissue samples and *n* = 12 TF samples) and B (*n* = 34 tumor tissue samples) (Fig. 1). Cluster A contained a high fraction of samples from patients with non-metastatic disease at diagnosis (M0), as well as the TF tissue samples, whereas samples from patients with metastasis at diagnosis (M1) were enriched in cluster B (*p* < 0.001). Cluster B tumors had larger tumor diameter (*p* < 0.001), higher morphological grade (*p* < 0.001), higher TNM stage (*p* < 0.001) and poorer outcome (Table 1, Fig. 2). There was no difference in gender or age at diagnosis between cluster A and B (Table 1).

**Table 1:** Clinicopathological parameters, methylation and genetic alterations and its relation to cluster status

	All Samples (*n* = 115)	Cluster A (*n* = 81)	Cluster B (*n* = 34)	*p*-value
Age (median ± SD)	65.0 ± 11.6	67.0 ± 11.5	63.0 ± 11.2	0.103^1^
Gender				
Male	66	42	24	0.064^2^
Female	49	39	10	
Tumor diameter (mm, median ± SD)	70.0 ± 41.6	55.0 ± 36.1	95.0 ± 38.5	< 0.001^1^
Morphological grade				
G1	16	16	0	< 0.001^2^
G2	46	40	6	
G3	34	20	14	
G4	18	5	13	
T Stage				
T1	50	47	3	< 0.001^2^
T2	25	17	8	
T3	39	17	22	
T4	1	0	1	
M stage				
M0	87	70	17	< 0.001^2^
M1	28	11	17	
TNM				
I	49	46	3	< 0.001^2^
II	14	12	2	
III	24	12	12	
IV	28	11	17	
Progress (*n* = 87)				
No	64	57	7	0.001^2^
Yes	23	13	10	
Follow Up Status				
Living	39	35	4	< 0.001^2^
Living with disease	5	3	2	
Dead in ccRCC	45	20	25	
Dead	26	23	3	
Average methylation (median ± SD)	0.3215 ± 0.0136	0.3196 ± 0.0105	0.3344 ± 0.0127	< 0.001^1^
Loss Chr 9p				
WT	73	62	11	< 0.001^2^
Loss	42	19	23	
Loss Chr 9q				
WT	75	62	13	< 0.001^2^
Loss	40	19	21	
Loss Chr 14q				
WT	76	60	16	0.005^2^
Loss	39	21	18	
Risk score (*n* = 114) [33]				
Low Risk	63	45	18	0.745^2^
High Risk	51	35	16	

Differences between groups are compared using ^1^ Mann-Whitney Test and ^2^ χ2-test

Cancer specific survival (pCSS) analysis confirmed the prognostic relevance of methylation cluster classification in ccRCC (pCSS_5yr_ 80% for cluster A vs 27% for cluster B, *p* < 0.001) (Fig. 2a). As previously known, the difference in CSS is large between patients with distant metastasis (M1, *n* = 28) at diagnosis and patients with non-metastatic disease (M0, *n* = 87) (pCSS_5yr_ 7% vs. 84%, *p* < 0.001) (Fig. 2b). We found no difference in pCSS_5yr_ in the M1 patients regardless of cluster status (pCSS_5yr_ 9% vs 6%, *p* = 0.840) (Fig. 2c), whereas cluster status clearly separated survival in the M0 patients (pCSS_5yr_ 92% vs 50%; *p* < 0.001) (Fig. 2c). Importantly, the cumulative incidence of progress (CIP) in M0 patients showed a significant difference between cluster A and B (*p* < 0.001), with lower incidence in cluster A patients (pCIP_5yr_ 15%) compared to cluster B patients (pCIP_5yr_ 63%) (Fig. 2d). However, methylation cluster status did not remain as an independent prognostic marker for neither CSS (*n* = 115) nor progression free survival (PFS) (*n* = 87) in multivariate Cox regression analysis including cluster status, TNM-stage, morphological grade, age and gender. In this analysis, only TNM stage, grade, and gender remained as independent markers (Additional file 8: Table S4 and Additional file 9: Table S5).

To relate cluster A/B status to previously defined prognostic panels in ccRCC, we classified the tumor samples based on the five CpG-site Risk classification panel described by Wei et al., (2015) [33]. Risk score could be calculated for 114 out of 115 tumor samples and were classified as Low (*n* = 63) or High risk (*n* = 51). There was no significant correlation between cluster A/B status and Wei Risk score (*p* = 0.745, Table 1). Risk classification according to Wei predicted CSS (pCSS_5yr_ 72% for Low risk vs 55% for High risk, *p* = 0.027) (Additional file 10: Figure S3A), but not as strong as genome-wide promoter methylation cluster classification (Fig. 2a). When stratifying patients into non-metastatic (*n* = 86) and metastatic (*n* = 28) groups according to Wei there was neither any statistical difference in CSS (pCSS_5yr_ 88% for Low risk vs 78% for High risk *p* = 0.113) in non-metastatic tumors nor between Low risk vs High risk in metastatic tumors (pCSS_5yr_ 0% vs 12%, *p* = 0.288, Additional file 10: Figure S3B). Further there was no difference in CIP between non-metastatic tumors with low risk versus high risk (pCIP_5yr_ 20 and 31% respectively, *p* = 0.099) (Additional file 10: Figure S3C).

### DNA methylation and genetic aberrations

The frequency of 12 common genetic aberrations in ccRCC as defined by Köhn et al. 2014 [9] (listed in Additional file 2: Table S3 and Additional file 5: Figure S2), were related to methylation status in the 115 ccRCCs. Among the 12 genomic regions, aberrations were found in 15 to 84% of the ccRCCs (Fig. 1, Additional file 2: Table S3, Additional file 5: Figure S2).

The most common genetic aberration in ccRCC is loss of chromosome 3p (including the VHL gene) [48]. In our cohort, 84% of the ccRCCs showed a loss of 3p, and deletions were similarly distributed in clusters A and B (Fig. 1, Additional file 2: Table S3). Loss of 9p (*p* < 0.001), 9q (*p* < 0.001) and 14q (*p* = 0.005) were more frequently observed in cluster B samples, whereas the other aberrations were similarly distributed among the clusters (Fig. 1, Table 1).

The chromosomal distribution of hyper- and hypomethylated CpGs was analysed in relation to the genomic aberrations in the 12 previously defined regions (Additional file 2: Table S3 and Additional file 6: Figure S4). Although genetic aberrations on chromosome 7p and 9q were associated with overrepresentation of hyper- and/or hypomethylated CpGs, there were no general enrichment of DM-CpGs within genomic regions harboring aberrations (Additional file 6: Figure S4). Importantly, loss of 3p which is frequently observed in ccRCC was not associated with significant accumulation of neither hyper- nor hypomethylated CpGs (Additional file 6: Figure S4).

**Fig. 3:**
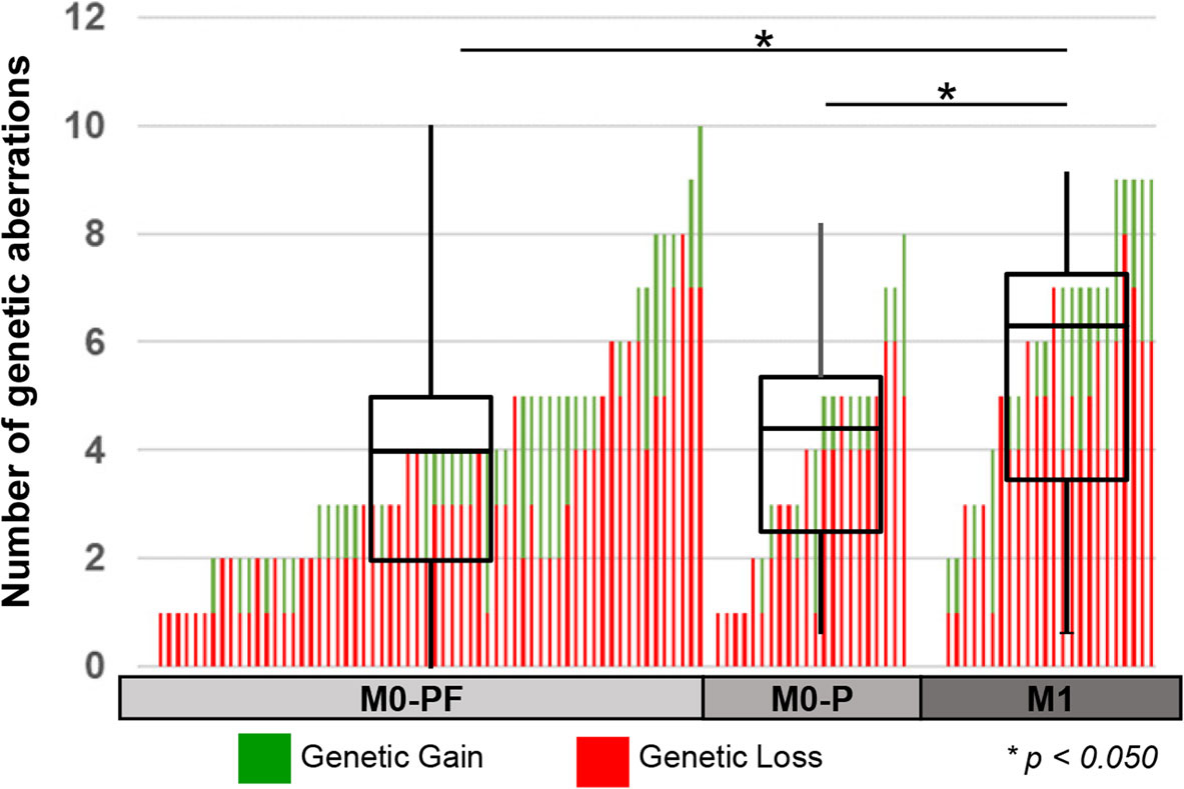
Distribution of genetic aberrations in ccRCC subgroups. Bar-plot showing number of genetic aberrations in individual samples. Red colour indicates genetic loss, and green colour genetic gain. Inserted boxplots show the median number of genetic aberrations and variations, in the M0-PF, M0-P, and M1 subgroups

We observed an intra-individual heterogeneity in the number of aberrations within the M0-PF (progress-free), M0-P (progress) and M1 (distant metastasis at diagnosis) group of patients (Fig. 3). M1 patients had generally more genomic aberrations compared with the M0-PF and M0-P patients (*p* = 0.024 and *p* = 0.050, respectively) (Fig. 3 and Table 2). The only genetic aberration that was more frequent in the M0-P compared to M0-PF patients, was the loss of 9q (43% vs. 20%, *p* = 0.031), which was even more frequent in the M1 patients (61%) (Table 2).

**Table 2:** Clinicopathological parameters, methylation and genetic alternations and its relation to ccRCC progression

		M0-PF (*n* = 64)	M0-P (*n* = 23)	M1 (*n* = 28)	*p*-value all groups of patients	*p*-value M0-PF vs. M0-P	*p*-value M0-PF vs. M1	*p*-value M0-P vs. M1
Age	(median ± SD)	68.5 ± 11.2	64.0 ± 12.7	63.5 ± 11.0	0.103^1^	0.165	0.093^3^	0.857^3^
Gender	Male	34	11	21	0.087^2^	0.663^2^	0.049^2^	0.046^2^
	Famale	30	12	7				
Tumor diameter (mm)	(median ± SD)	50.0 ± 30.7	90.0 ± 45.7	100.0 ± 35.7	< 0.001^1^	0.001^3^	< 0.001^3^	0.107^3^
Morphological grade	G1	14	2	0	< 0.001^2^	0.006^2^	< 0.001^2^	0.199^2^
	G2	31	8	7				
	G3	18	8	8				
	G4	1	5	12				
T Stage	T1	42	7	1	< 0.001^2^	0.010^2^	< 0.001^2^	0.054^2^
	T2	11	6	8				
	T3	11	10	18				
	T4	0	0	1				
TNM	I	42	7	0	< 0.001^2^	0.001^2^	< 0.001^2^	< 0.001^2^
	II	11	3	0				
	III	11	13	0				
	IV	0	0	28				
Follow Up Status	Alive	38	1	0	< 0.001^2^	< 0.001^2^	< 0.001^2^	0.126^2^
	Alive, disease	0	4	1				
	Dead, ccRCC	0	18	27				
	Dead	26	0	0				
Cluster	A	57	13	11	< 0.001^2^	0.001^2^	< 0.001^2^	0.220^2^
	B	7	10	17				
PMC status	Low	36	4	–	–	0.001	–	–
	High	28	19					
DM-CpGs, tot. Number	(median ± SD)	9191 ± 4779	10,460 ± 3698	12,996 ± 4448	0.001^1^	0.227^3^	0.005^3^	0.130^3^
Hypermethylated CpGs	(median ± SD)	5826 ± 2820	7617 ± 2990	8233 ± 448	0.015^1^	0.025^3^	0.001^3^	0.150^3^
Hypomethylated CpGs	(median ± SD)	2810 ± 3173	2843 ± 2990	2688 ± 4249	0.970^1^	0.810^3^	0.905^3^	0.910^3^
Mitotic Age	(median ± SD)	0.16 ± 0.05	0.18 ± 0.05	0.18 ± 0.06	0.047^1^	0.132^3^	0.021^3^	0.538^3^
Loss 1p	WT	42	19	18	0.272^2^	0.127^2^	0.901^2^	0.145^2^
	Loss	22	4	10				
Loss 3p	WT	11	3	4	0.288^2^	0.643^2^	0.729^2^	0.898^2^
	Loss	53	20	24				
Loss 3q	WT	45	20	21	0.995^2^	0.115^2^	0.646^2^	0.285^2^
	Loss	19	3	7				
Gain 5q	WT	30	11	13	0.054^2^	0.938^2^	0.969^2^	0.921^2^
	Gain	34	12	15				
Loss 6q	WT	50	22	26	0.027^2^	0.056^2^	0.086^2^	0.673^2^
	Loss	14	1	2				
Gain 7p	WT	51	21	17	0.061^2^	0.206^2^	0.057^2^	0.013^2^
	Gain	13	2	11				
Gain 7q	WT	51	21	18	0.598^2^	0.206^2^	0.116^2^	0.024^2^
	Gain	13	2	10				
Loss 8p	WT	47	15	18	0.002^2^	0.455^2^	0.375^2^	0.945^2^
	Loss	17	8	10				
Loss 9p	WT	49	13	11	0.002^2^	0.069^2^	0.001^2^	0.220^2^
	Loss	15	10	17				
Loss 9q	WT	51	13	11	0.001^2^	0.031^2^	< 0.001^2^	0.220^2^
	Loss	13	10	17				
Loss 10q	WT	47	20	20	0.361^2^	0.186^2^	0.842^2^	0.180^2^
	Loss	17	3	8				
Loss 14q	WT	48	13	15	0.076^2^	0.097^2^	0.042^2^	0.833^2^
	Loss	16	10	13				
Tot. number of alterations	(median ± SD)	4 ± 2	4 ± 2	6 ± 3	0.157^1^	0.934^3^	0.024^3^	0.050^3^

Differences between groups are compared using ^1^Kruskall-Wallis Test, ^2^χ2-test and ^3^Mann-Whitney Test

**Fig. 4:**
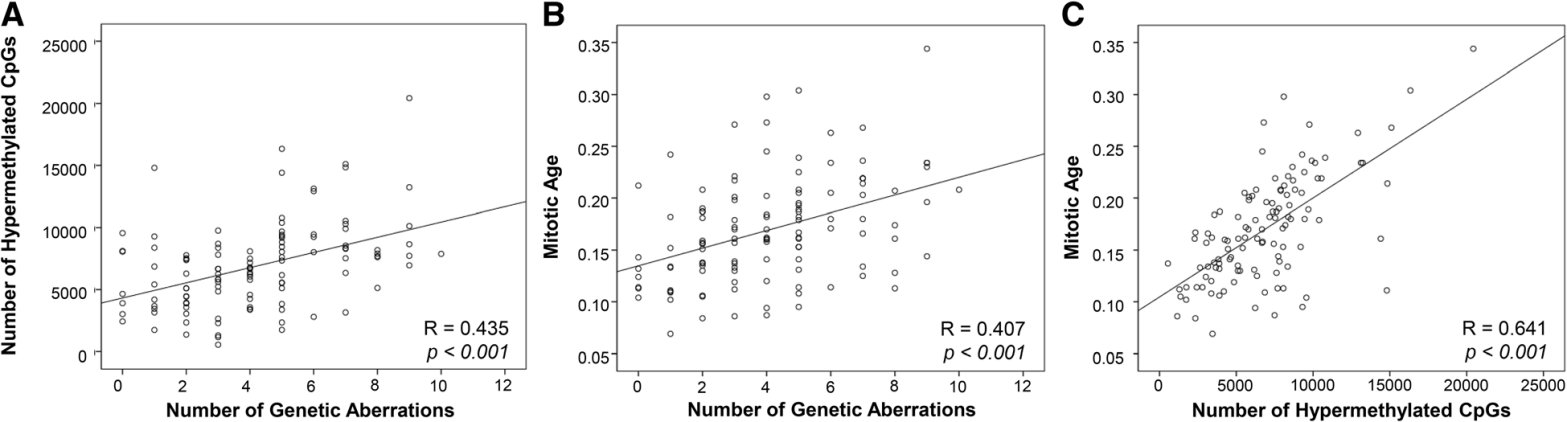
Correlations between number of hypermethylated CpGs and number of genetic aberrations, and predicted mitotic age. Scatterplots showing correlation between (**a**) number of hypermethylated CpGs and number of genetic aberrations; (**b**) mitotic age and number of genetic aberrations and (**c**) mitotic age and number of hypermethylated CpGs. Bivariate correlation and *p*-values are presented

Genetic and epigenetic aberrations may accumulate during extended replication. In order to evaluate such possible associations in ccRCC, we analysed the number of hyper- and hypomethylated CpG sites in relation to number of genetic alterations and estimated mitotic age of the tumor samples. The number of genetic aberrations were positively correlated to number of hypermethylated CpGs (*p* < 0.001), but not to the number of hypomethylated CpGs in promoters (Fig. 4a, Additional file 11: Figure S5A). The number of genetic aberrations and number of hypermethylated CpGs were both strongly positively associated with estimated mitotic age [41] (R = 0.407, and R = 0.641, respectively, *p* < 0.001) (Fig. 4b-c), but there was no correlation between number of hypomethylated CpGs and mitotic age (Additional file 11: Figure S5B).

### Methylation profiles associated with progression of disease

Since cluster status was of prognostic relevance in M0 patients, we focused the analysis on identifying methylation alterations associated with clinical progress.

M0-PF, M0-P and M1 patients showed gradually larger median tumor diameter (*p* < 0.001), higher TNM stage (*p* < 0.001), higher morphological grade (*p* < 0.001), increased number of hypermethylated CpGs (*p* = 0.015), higher mitotic age (*p* = 0.047), and a higher correlation to cluster B status (*p* < 0.001) (Table 2). The M0-PF and M0-P groups differed significantly in all these clinicopathological parameters (except for mitotic age) (Table 2). In contrast, the M0-P and M1 groups differed in TNM (*p* < 0.001) and gender distribution only (*p* = 0.046) (Table 2).

**Fig. 5:**
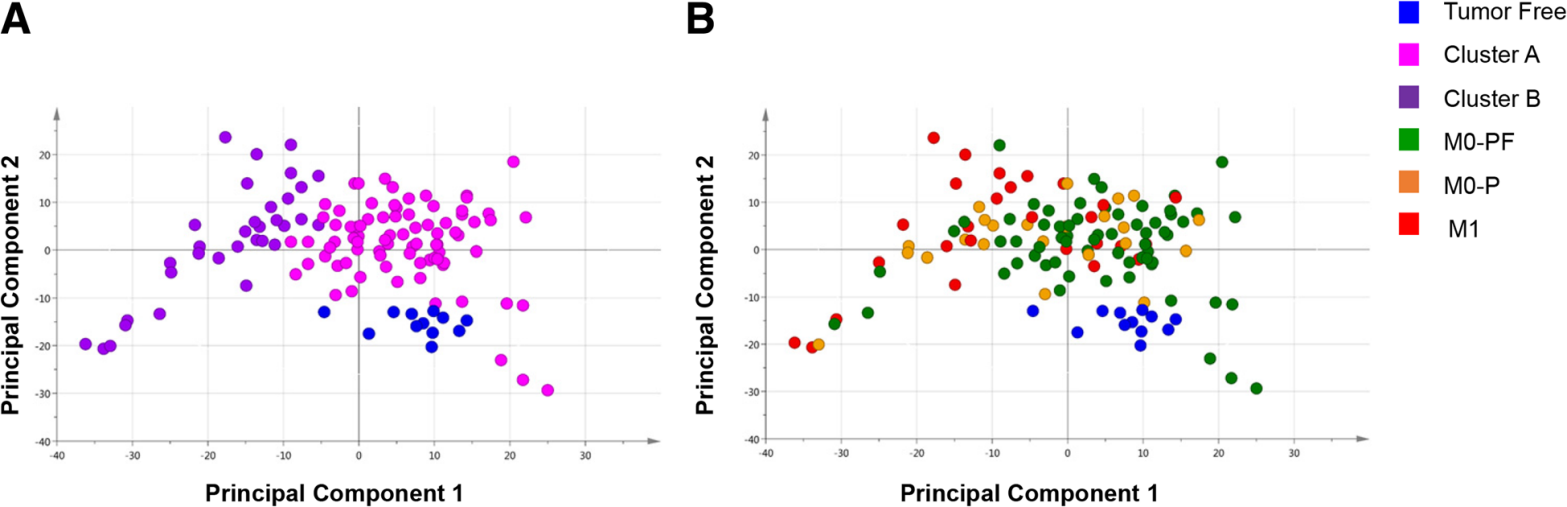
Principal component analysis (PCA) of promoter associated DNA methylation. Average methylation of 155,931 promoter associated CpGs on 115 ccRCC and 12 tumor free (TF) adjacent tissue samples are plotted, after centring average beta values, using the first two principal components. All samples are represented by a dot and subgroups are highlighted according to (**a**) TF-tissue samples, cluster **a** and cluster **b**; (**b**) TF-tissue samples, ccRCC without distant metastasis at diagnosis that did not progress (M0-PF), ccRCC without distant metastasis at diagnosis that did progress (M0-P) and ccRCC with distant metastasis at diagnosis (M1)

With the aim to distinguish TF, M0-PF, M0-P and M1 subgroups of patients based on the promoter associated methylation profiles, we used principal component analysis (PCA) (Fig. 5). All ccRCC and TF samples were included in the PCA, and different sample categories were highlighted to identify similarities and/or differences. TF tissue samples showed a homogenous methylation pattern (Fig. 5a-b), and cluster A and cluster B patients could be separated (Fig. 5a). However, M0-PF, M0-P and M1 patients could not be clearly separated by PCA since their methylation patterns were partly overlapping (Fig. 5b).

**Fig. 6:**
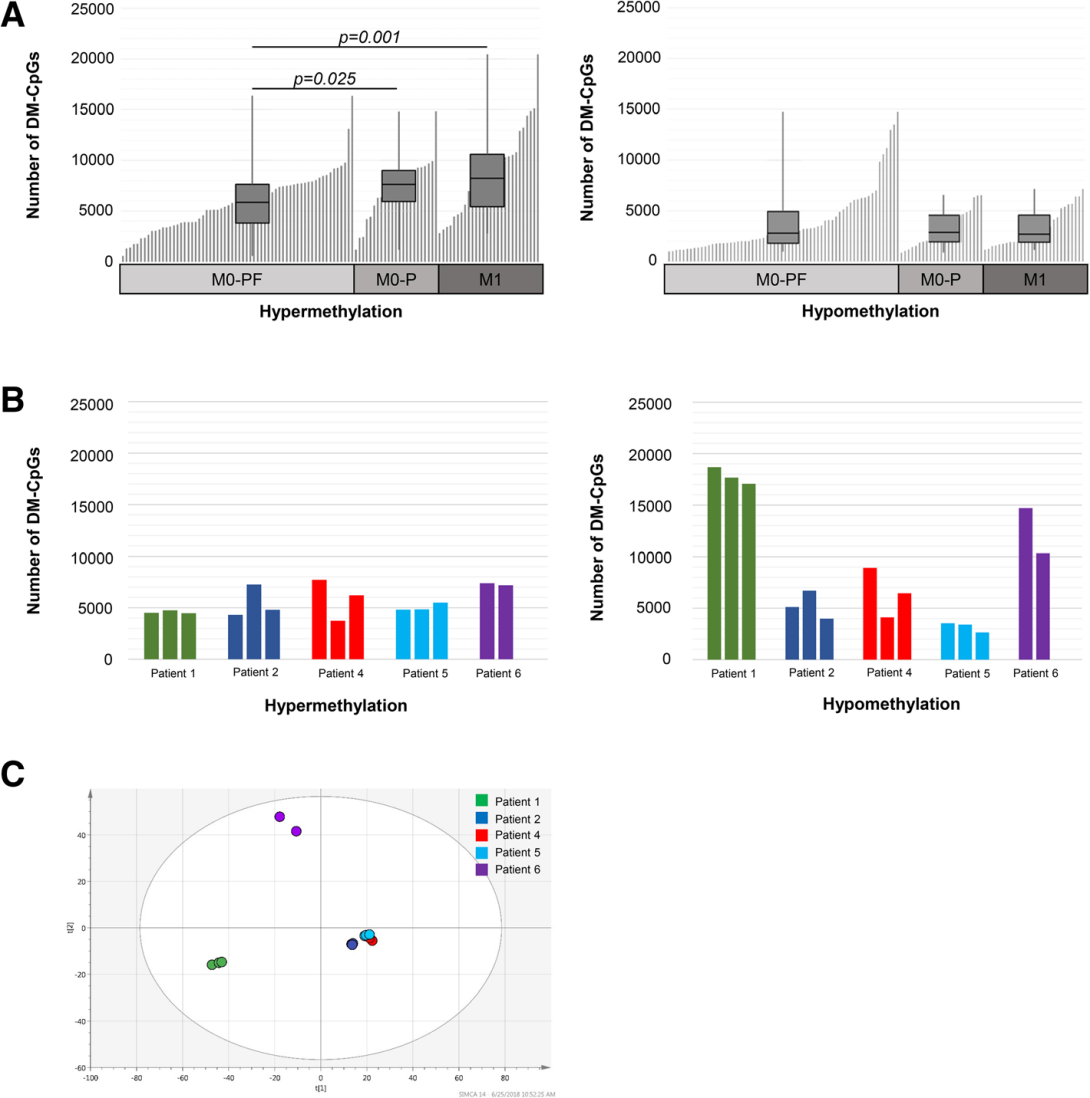
Differently methylated CpG sites (CpGs) and its distribution over clear cell renal cell carcinoma (**a**) Bar-plot of number of hyper- (left panel) and hypomethylated (right panel) CpGs, within promoter regions of individual samples in the M0-PF, M0-P and M1 ccRCC groups. Inserted boxplots show number of hyper- and hypomethylated CpGs and its variation within the subgroups. Differences between groups were analysed using Mann-Whitney U-test. **b** Bar-plot for five ccRCC patients (number 1 through 6) represented by two to three pieces from the same tumor, showing the number of hypermethylated (left panel) and hypomethylated (right panel) promoter associated CpGs; **c**) PCA analysis of the first two principal components of the average methylation of promoter associated CpGs in multiple samples from five ccRCC patients. All samples are represented by a dot and each individual is highlighted according to legend in figure

Instead, we aimed to identify specific CpGs associated with progress after primary treatment. DM-CpGs were identified in each tumor sample using the average methylation for TF tissue samples as a reference. Large inter-individual variations in the number of hyper/hypomethylated DM-CpGs was observed in the tumor samples (Fig. 6a). The number of hypermethylated DM-CpGs were significantly higher in the M0-P and M1 patients compared with M0-PF (*p* = 0.025 and 0.001, respectively) (Fig. 6a, left chart). The number of hypomethylated DM-CpGs did not differ significantly between the groups of patients (Fig. 6a, right chart).

To further dissect possible variations in methylation profiles at intra-tumoral level, we analysed multiple samples taken from various parts of the tumor in five individuals (Additional file 12: Table S6). We found that samples from the same tumor generally had similar methylation profiles (R^2^ correlations 0.97 to 0.99; *p* ≤ 0.001). Accordingly, the number of hyper- and hypomethylated CpGs were similar (Fig. 6b), and the individuals´ samples clustered together in PCA-analysis (Fig. 6c).

**Fig. 7:**
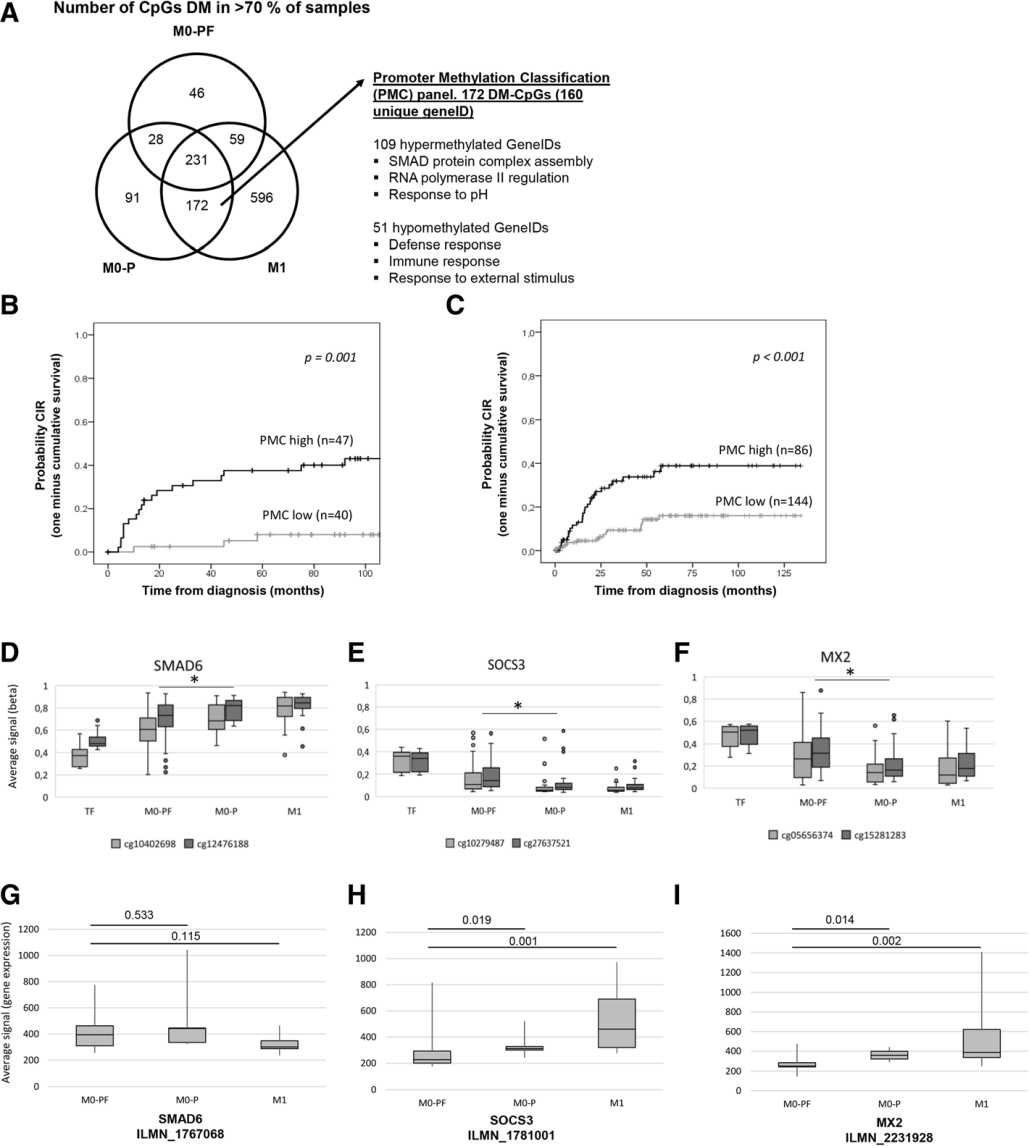
Identification of CpGs associated with progress/metastasis in ccRCC. **a** Venn-diagram showing overlap of CpGs that were differently methylated compared with TF tissue, in more than 70% of samples in each ccRCC group; M0-PF; M0-P and M1. The 172 commonly DM-CpGs in the M0-P and M1 groups were defined as a Promoter Methylation Classification (PMC) panel. Selected GO-terms associated with hyper- and hypomethylated genes in PMC panel are listed. **b**-**c** Kaplan-Meier Cumulative incidence of progress (CIP) (**b**) in 87 non-metastatic ccRCC patients (**c**) in 230 non-metastatic ccRCC patients in the validation TCGA-KIRC cohort. **d**-**f** Box-plots of selected DM-CpGs in promoter regions of genes associated with progress (**d**) SMAD6 (**e**) SOCS3 and (**f**) MX2. *p < 0.05. **g**-**i** Box-plots of mRNA levels for (**g**) SMAD6, (**h**) SOCS3 and (**i**) MX2. Differences in methylation and mRNA levels between groups were analysed using Mann-Whitney U-test

DNA methylation alterations have been described as early events in tumor progression. Therefore, we focused on common DM-CpGs in the M0-P and M1 samples, with potential mQTLs excluded [40], which might represent epigenetic alterations of relevance for the formation of metastasis. CpGs that showed either hyper- or hypomethylation in at least 70% of samples in the M0-PF, M0-P and M1 groups of patients were selected and combined in a Venn diagram (Fig. 7a). Most of the DM-CpGs in the M0-PF patients were also present in the M0-P and M1 patients, indicating that methylation alterations accumulated during progression and was not dominated by stochastic events. Only a low percentage of DM-CpGs were unique for M0-PF and M0-P (13 and 17% respectively) whereas M1 had 56% unique DM-CpGs. 172 DM-CpGs were common for the M0-P/M1 patient groups (121 hyper- and 51 hypo-methylated) (Fig. 7a). These 172 DM-CpGs were defined as a Promoter Methylation Classifier (PMC) panel, and was used to classify samples as PMC low and high as described in the materials and methods section. In the non-metastatic tumors of our cohort, 40 samples were classified as PMC low and 47 samples as PMC high. The pCIP_5yr_ survival analysis showed poorer outcome in the PMC high subgroup (PMC low pCIP_5yr_ 8% vs. PMC high pCIP_5yr_ 38%, *p* = 0.001; Fig. 7b). The PMC panel (PMC high Hazard ratio (HR) 4.4 (1.3–15.8)) and TNM stage (TNM III HR 3.9 (1.4–11.0)) remained significant prognostic markers for PFS in a Cox regression analysis including PMC status, TNM, morphological grade, age and gender (Additional file 13: Table S7). The prognostic relevance of PMC was confirmed in 230 ccRCC tumor samples from the TCGA-KIRC data set (PMC low (*n* = 144) pCIP_5yr_ 16% vs. PMC high (*n* = 86) pCIP_5yr_ 39%, *p* < 0.001; Fig. 7c).

The functional relevance of the PMC associated genes were analysed for the most significant gene ontology categories (GO terms) (Fig. 7a, Additional file 7: Table S8). The commonly hypermethylated genes were enriched for GO terms including SMAD protein complex assembly, RNA polymerase II regulation and response to pH (Fig. 7a). The hypomethylated genes were enriched for GO terms associated with external stimulus-, immune- and defence- response (Fig. 7a). Three genes were of special interest with regard to ccRCC progress; i.e. SMAD family member 6 (SMAD6, CpG cg10402698 and cg12476188), Suppressor of cytokine signaling 3 (SOCS3, CpG cg10279487 and cg27637521) and MX dynamin like GTPase 2 (MX2, CpG cg05656374 and cg15281283). These genes showed significant altered methylation levels between TF/M0-PF and the M0-P/M1 samples in more than one promoter associated CpG site (Fig. 7d-f).

Methylation alterations in these genes were analysed in relation to mRNA gene expression alterations in 28 samples with available RNA (M0-PF = 13; M0-*P* = 5 and M1 = 10). A significant increased gene expression was observed in M0-P and M1 samples compared to M0-PF samples in the SOCS3 (ILMN_1781001; *p* = 0.019 and 0.001) and MX2 (ILMN_2231928; *p* = 0.014 and 0.002) genes, whereas no significant difference in expression for SMAD6 (ILMN_1767068) was seen (Fig. 7g-i).

## Discussion

The aim with this study was to evaluate if genome-wide promoter associated methylation classification can be used as a prognostic tool to identify patients with non-metastatic ccRCC at risk for disease progression. These patients may benefit from alternative therapy approaches such as adjuvant therapy and/or more intense follow-up.

Cluster analysis of 155,931 promoter associated CpG sites divided the 115 ccRCCs into two groups (clusters A and B), where the less methylated ccRCC samples in cluster A clustered together with TF samples. Cluster B status associated with higher average promoter methylation, and this group contained a high fraction of metastasized tumors and patients with local disease who later progressed. This confirmed our previous finding of poor prognosis associated with higher promoter methylation status in ccRCC [34]. Importantly, our current study showed that the prognostic relevance of DNA methylation was limited to M0 patients since the prognosis was very poor regardless of methylation status for patients with metastasized ccRCC at diagnosis.

In order to relate our data with previously described DNA methylation panels for risk stratification in ccRCC, we applied the five CpG-site risk panel defined by Wei et al., (2015) on our cohort [33]. We found no significant association between cluster status and Risk group classification. The Wei Risk score could neither separate the survival nor the progress prognosis in non-metastatic tumors, but was predictive for CSS when including the metastatic tumors.

To predict incidence of progress in non-metastatic patients, genome-wide promoter associated cluster analysis seems to be more efficient than risk stratification according to the Wei Risk score restricted to five CpG sites .

The previously described CIMP panel by Arai et al., (2012) could not be applied on our cohort since not all CpGs in the CIMP panel were present in the Infinium HumanMethylation450K array used in our study [31]. However, the Arai CIMP classification has previously shown that high methylation status was associated with poor prognosis, which is in line with our data.

Genomic aberrations are commonly observed in ccRCC and we performed an integrated analysis of methylation status and genomic aberrations in order to identify potential correlations. We used raw data from the methylation arrays to determine genomic aberrations in twelve regions previously defined as harboring common changes (loss or gain) in a subset of our cohort of ccRCC patients [9]. Using methylation arrays to identify genomic aberrations might introduce systematic bias due to segmentation at the ends of several chromosomes. However, the correlation between the identified genetic aberrations by methylation array and SNP-array analysis revealed comparable results in the subset of samples analysed by both techniques. The number of identified genetic alterations were likely underestimated since we focused the analysis on previously defined regions with aberrations [9]. Also, the number of DM-CpGs might be underestimated since we used histological normal tumor-adjacent tissue from a kidney with ccRCC as reference samples. Methylation differences have been reported between histological normal tumor-adjacent tissue and tissue taken from healthy individuals [31].

There was a significantly higher frequency of deletions of 9p, 9q and 14q in cluster B tumors, and these three genetic aberrations have been associated with poorer outcome in ccRCC [7–11, 49, 50]. Importantly, we did not find a general increased number of DM-CpGs in regions frequently lost or gained. Loss of chromosome 9q was the only genetic aberration that was more common in M0-P (and M1) patients compared to M0-PF patients. Previous studies have shown significant correlation of loss of chromosome 9q to both histological grade and TNM stage [9, 49, 50] as well as to poor outcome [9]. Patients with 9q loss also showed an enriched number of DM-CpGs within this region. The possible contribution of epigenetic alterations within this region, to poor prognosis, has to be further evaluated.

However, we observed a significant positive correlation of total number of hypermethylated DM-CpGs and total number of genetic aberrations, supporting previous results as shown in a review by Arai and Kanai [51]. In that study a correlation between number of clones with CNVs and total number of DM-CpGs was shown in a subset of ccRCC samples with high methylation levels. The correlation between number of hypermethylated CpGs, number of genetic aberrations, and predicted mitotic age indicates an accumulation of alterations associated with number of cell divisions. Correlation between genetic and epigenetic alterations was shown previously in both chronic lymphatic leukemia [52] and in breast cancer cell lines [53], but less is known about correlations with mitotic age.

The fact that genome-wide promoter methylation cluster status separated the survival time of patients with non-metastatic disease at diagnosis, made us focus on identifying the specific methylation profiles associated with progress. Initially, PCA was used to investigate whether patient groups with different outcome could be separated based on general promoter methylation patterns. This analysis showed overlapping and heterogeneous methylation patterns within the M0-PF, M0-P and M1 groups, in contrast to homogenous methylation patterns within the TF samples. Also, patients with similar outcome showed large inter-individual variations in methylation patterns. This cannot be explained by intra-individual tumor heterogeneity since multiple samples taken within the same tumor showed similar methylation profiles.

By recognizing the commonly DM-CpGs in the M0-P and M1 samples, we identified a Promoter Methylation Classifier (PMC) consisting of 172 CpGs associated with progress. Classification of non-metastatic patients in PMC high/low subgroups showed strong prognostic relevance for progress in our cohort as well as in the validation TCGA-KIRC cohort. Importantly, the PMC panel remained a significant prognostic marker for progression free survival in a Cox regression analysis including PMC status, TNM, morphological grade, age and gender.

The genes in the PMC panel were associated with cellular processes including SMAD protein complex assembly and immune response. Genes of special interest previously associated with various types of cancer where the SMAD6 and SOCS3 genes coupled to aggressive kidney cancer [54–56], and the MX2 gene with suggested role in melanoma pathogenesis [57]. Interestingly these genes were differently methylated in the M0-P group, compared with TF and M0-PF samples and a significant difference in mRNA levels was observed for the SOCS3 and MX2 genes. These findings indicate a functional relevance of the methylation alterations in ccRCC pathogenesis but needs to be confirmed in larger samples cohorts.

In a systematic review by Joosten et al., 2017, a number of DNA methylation studies in ccRCC were summarized [30] and the need for validation of identified prognostic markers was claimed. We could not confirm the prognostic relevance of the previously defined Wei risk score (based on five CpG sites) in our cohort. Instead, we could confirm the prognostic relevance of genome-wide promoter methylation cluster analysis (Cluster A/B, > 150 K CpGs), suggesting that larger panels are probably more robust. However, in contrast to genome-wide clustering, a defined set of CpGs (or genes) is likely more clinically suitable. In this study, we defined a PMC panel consisting of 172 CpGs, which was a strong prognostic marker for non-metastatic patients in both our cohort and in the validation cohort. Modern bioinformatics tools that combines DNA methylation classification with clinical prognostic markers is an important next step to implement epigenetic analysis in clinical practice.

## Conclusion

Genome-wide promoter-associated DNA methylation associated significantly with genetic aberrations, cellular mitotic age, and clinical parameters, including follow up status. We defined a Promoter Methylation Classification (PMC) panel, including genes of potential relevance for tumor progression. The PMC panel predicted progression free survival in non-metastatic ccRCC patients, which was confirmed in the independent TCGA-KIRC cohort. DNA methylation status has the potential to identify non-metastatic patients with high risk of recurrence already at diagnosis. These high-recurrence risk patients may benefit from alternative therapy approaches and more intense follow-up.

## Additional files

Additional file 1: **Table S1.** Filtration steps in the HumanMeth450K arrays. (PDF 79 kb)

Additional file 2: **Table S3.** Analyzed genomic regions for CNV in 115 ccRCC samples. (PDF 74 kb)

Additional file 3: **Figure S1.** Single nucleotide polymorphism (SNP) analysis. SNP analysis of 65 genotyping probes on the HumanMethylation450K array to confirm patient identity of multiple samples taken from the same tumor. (PDF 256 kb)

Additional file 4: **Table S2.** Comparison of CNV results gained from HumanMethylation450K and HumanCytoSNP-12 arrays in 57 ccRCC samples. (PDF 102 kb)

Additional file 5: **Figure S2.** Copy number variation (CNV) analysis. CNV analysis of twelve chromosome regions previously identified to be altered in ccRCC. The analyzed region is marked in grey. (PDF 205 kb)

Additional file 6: **Figure S4.** Distribution of hyper- and hypomethylated CpGs in patients with or without specific genomic aberrations. Percentage of (A) hypermethylated and (B) hypomethylated CpGs in the genomic aberration regions associated with ccRCC defined in Additional file 2: Table S3. * = Bonferroni adjusted *p*-value < 0.05. (PDF 75 kb)

Additional file 7: **Table S8.** Top 20 most significant GO Terms for GeneIDs either hyper- or hypomethylated in M0-P and M1 tumor samples. (PDF 54 kb)

Additional file 8: **Table S4.** Cox’s proportional hazard regression analysis for cancer specific survival (CSS) in 115 ccRCC samples. (PDF 108 kb)

Additional file 9: **Table S5.** Cox’s proportional hazard regression analysis for progress free survival (PFS) in 87 M0 ccRCC samples. (PDF 108 kb)

Additional file 10: **Figure S3.** Survival analysis based on Risk Score at diagnosis. Kaplan-Meier cancer specific survival analysis (pCSS) in 114 ccRCC patients in relation to (A) Wei Risk Score at diagnosis (B) a combination of Wei Risk Score and presence of distant metastasis at diagnosis. (C) Cumulative incidence of progress (CIP) analysis in 86 non-metastatic (M0) ccRCC patients in relation to Wei Risk Score at diagnosis. Log-rank *p*-values are presented. (PDF 79 kb)

Additional file 11: **Figure S5.** Correlations between number of hypomethylated CpGs and number of genetic aberrations and predicted mitotic age. Scatterplots showing correlation between (A) number of hypomethylated CpGs and number of genetic aberrations; (B) mitotic age and number of hypomethylated CpGs. Bivariate correlation and *p*-values are presented. (PDF 74 kb)

Additional file 12: **Table S6.** Clinicopathological parameters for tumors included in the heterogeneity analysis. (PDF 65 kb)

Additional file 13 **Table S7.** Cox’s proportional hazard regression analysis for progress free survival (PFS) in 87 M0 ccRCC samples. (PDF 108 kb)

## Abbreviations

ccRCC: clear cell renal cell carcinoma
CpG: cytosine-phosphate-guanine
DM-CpG: differently methylated CpG site
DMG: differently methylated gene
M0-P: non-metastatic ccRCC at diagnosis that later progress
M0-PF: non-metastatic ccRCC at diagnosis and progress-free at follow up
M1: metastatic ccRCC at diagnosis
PMC: promoter methylation classifier
TF: tumor free
TNM: tumor-node-metastasis
