# Supplementary material for: DNA methylation associates with survival in non-metastatic clear cell renal cell carcinoma
Source: BMC Cancer. 2019 Jan 14;19:65. doi: 10.1186/s12885-019-5291-3 (PMC6332661; doi:10.1186/s12885-019-5291-3)
Supplement: Supplementary file 1 — Table S1. Filtration steps in the HumanMeth450K arrays. (PDF 79 kb) [file 12885_2019_5291_MOESM1_ESM.pdf]

Additional Table 1

Filtration steps	Unique CpGs	Unique genes
1. Annotation (updated)	483214	20972
2. Excluding of chr X and Y	471657	20022
3. Exclucing CpGs less than 3 bp from a snp, multi-hit	433954	19799
4. Excluding CpGs with a detection p-value greater than 0.05 or less than 3 nbeads in any sample	396543	19758
4. Excluding CpGs located outside promoter region	155931	19385
