# Supplementary material for: DNA methylation associates with survival in non-metastatic clear cell renal cell carcinoma
Source: BMC Cancer. 2019 Jan 14;19:65. doi: 10.1186/s12885-019-5291-3 (PMC6332661; doi:10.1186/s12885-019-5291-3)
Supplement: Supplementary file 2 — Table S3. Analyzed genomic regions for CNV in 115 ccRCC samples. (PDF 74 kb) [file 12885_2019_5291_MOESM2_ESM.pdf]

Additional Table 3

Genetic abberation	Percentage of tumors (n=115 ccRCC)	Minimum Region
1p, loss	32	23,334,666 – 32,100,024
3p, loss	84	tel – 46,765,401
3q, loss	25	cen – 97,267,562
5q, gain	53	172,012,494 – tel
6q, loss	15	152,878,353 – tel
7p, gain	23	Whole arm
7q, gain	22	Whole arm
8p, loss	30	12,653,559 – 27,797,474
9p, loss	37	tel – 28,013,465
9q, loss	35	130,719,998 – 138,443,132
10q, loss	25	85,932,576 – 90,524,678
14q, loss	37	69,560,017 – 91,189,113
