# Supplementary figures and images for: DNA methylation associates with survival in non-metastatic clear cell renal cell carcinoma
Source: BMC Cancer. 2019 Jan 14;19:65. doi: 10.1186/s12885-019-5291-3 (PMC6332661; doi:10.1186/s12885-019-5291-3)

Additional Figure 1

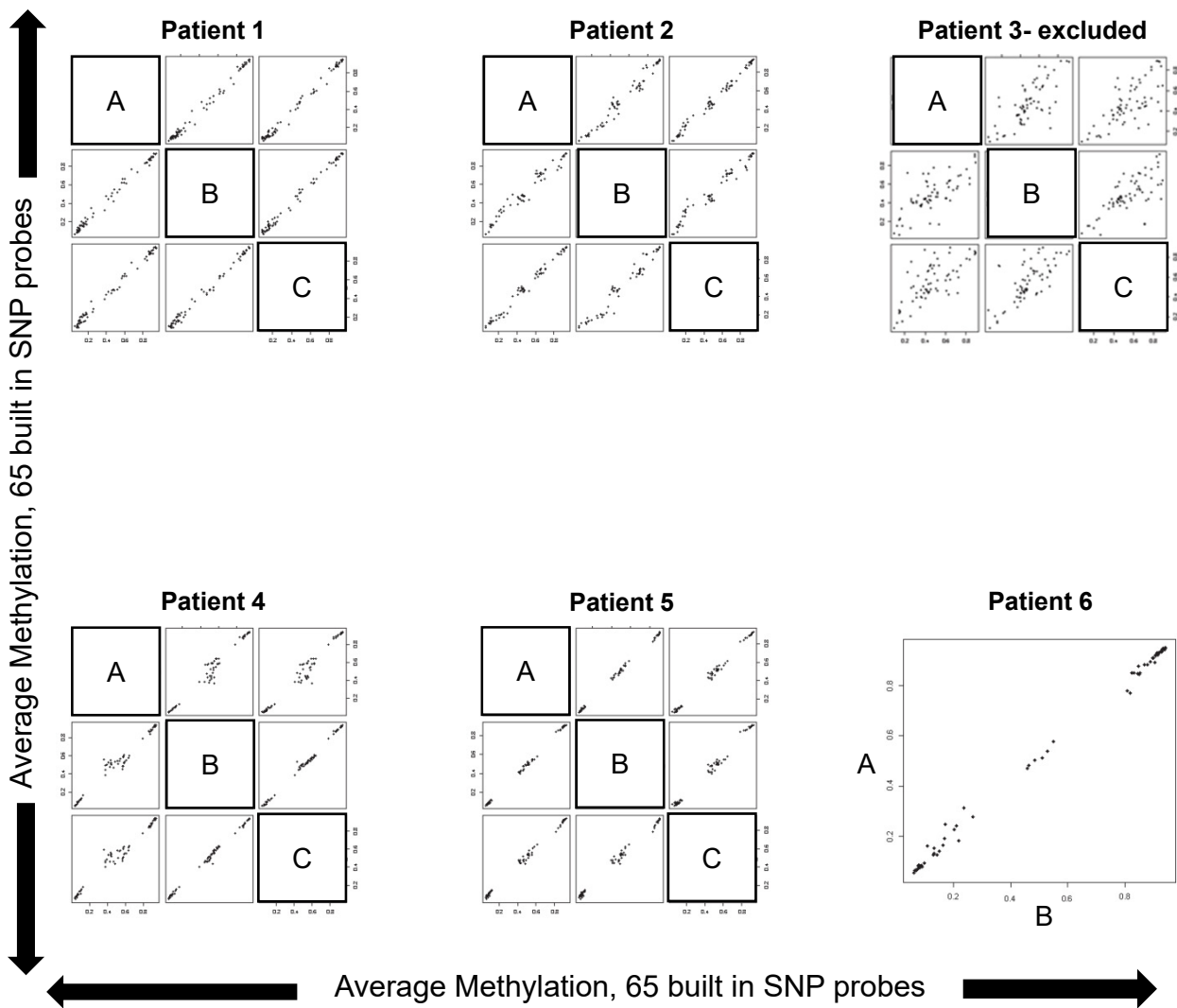

Supplement: Supplementary file 3 — Figure S1. Single nucleotide polymorphism (SNP) analysis. SNP analysis of 65 genotyping probes on the HumanMethylation450K array to confirm patient identity of multiple samples taken from the same tumor. (PDF 256 kb) [file 12885_2019_5291_MOESM3_ESM.pdf]

Additional Figure 2

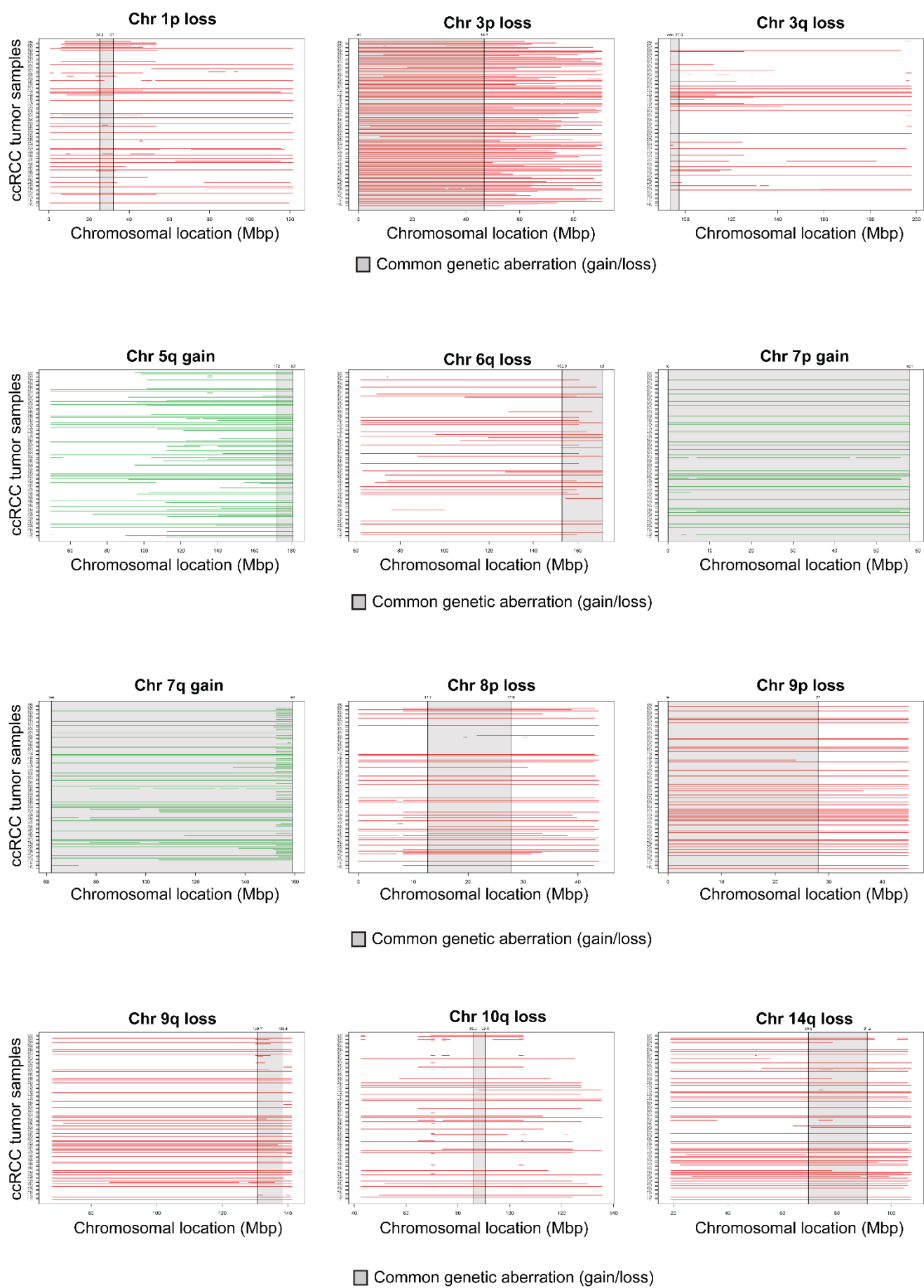

Supplement: Supplementary file 5 — Figure S2. Copy number variation (CNV) analysis. CNV analysis of twelve chromosome regions previously identified to be altered in ccRCC. The analyzed region is marked in grey. (PDF 205 kb) [file 12885_2019_5291_MOESM5_ESM.pdf]

Additional Figure 4

A

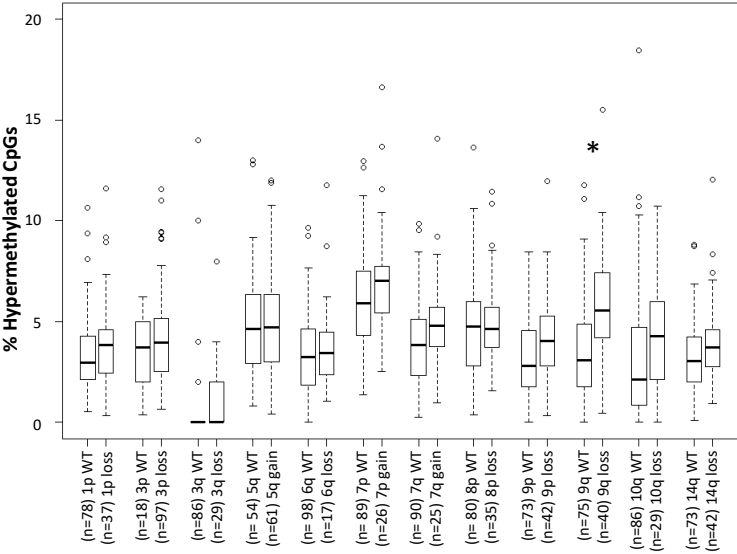

B

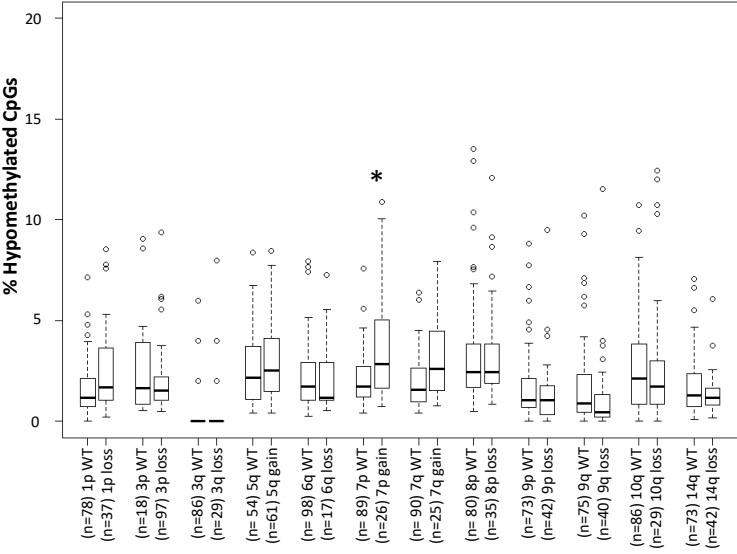

Supplement: Supplementary file 6 — Figure S4. Distribution of hyper- and hypomethylated CpGs in patients with or without specific genomic aberrations. Percentage of (A) hypermethylated and (B) hypomethylated CpGs in the genomic aberration regions associated with ccRCC defined in Additional file 2: Table S3. * = Bonferroni adjusted p-value < 0.05. (PDF 75 kb) [file 12885_2019_5291_MOESM6_ESM.pdf]

Additional Figure 3

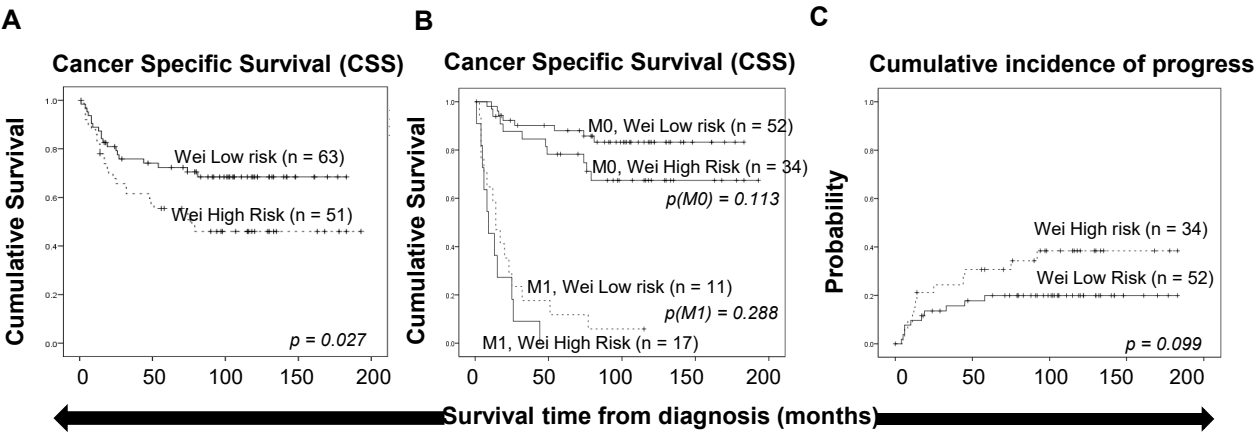

Supplement: Supplementary file 10 — Figure S3. Survival analysis based on Risk Score at diagnosis. Kaplan-Meier cancer specific survival analysis (pCSS) in 114 ccRCC patients in relation to (A) Wei Risk Score at diagnosis (B) a combination of Wei Risk Score and presence of distant metastasis at diagnosis. (C) Cumulative incidence of progress (CIP) analysis in 86 non-metastatic (M0) ccRCC patients in relation to Wei Risk Score at diagnosis. Log-rank p-values are presented. (PDF 79 kb) [file 12885_2019_5291_MOESM10_ESM.pdf]

Additional Figure 5

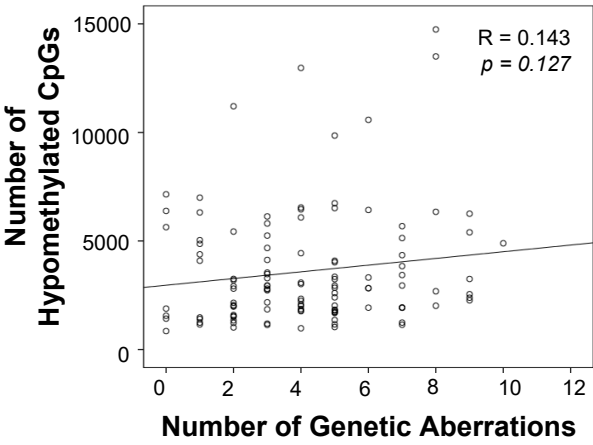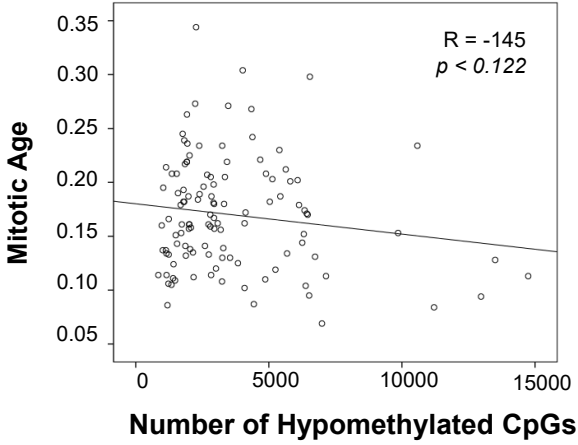

Supplement: Supplementary file 11 — Figure S5. Correlations between number of hypomethylated CpGs and number of genetic aberrations and predicted mitotic age. Scatterplots showing correlation between (A) number of hypomethylated CpGs and number of genetic aberrations; (B) mitotic age and number of hypomethylated CpGs. Bivariate correlation and p-values are presented. (PDF 74 kb) [file 12885_2019_5291_MOESM11_ESM.pdf]
