# Supplementary material for: DNA methylation associates with survival in non-metastatic clear cell renal cell carcinoma
Source: BMC Cancer. 2019 Jan 14;19:65. doi: 10.1186/s12885-019-5291-3 (PMC6332661; doi:10.1186/s12885-019-5291-3)
Supplement: Supplementary file 4 — Table S2. Comparison of CNV results gained from HumanMethylation450K and HumanCytoSNP-12 arrays in 57 ccRCC samples. (PDF 102 kb) [file 12885_2019_5291_MOESM4_ESM.pdf]

Additional Table 2

SNP analysis (n = 57) HumanMethylation450 BeadChip			SNP analysis (n = 57) HumanCytoSNP-12 v2.1			
			**WT**	**Loss/Gain**	Cohen's kappa	p-value[1]
	**1p**	WT<br>Loss	36<br>7	5<br>9	0.458	0.001
	**3p**	WT<br>Loss	5<br>3	4<br>45	0.516	< 0.001
	**3q**	WT<br>Loss	41<br>2	2<br>12	0.811	< 0.001
	**5q**	WT<br>Gain	24<br>7	2<br>24	0.687	< 0.001
	**6q**	WT<br>Loss	39<br>2	7<br>9	0.568	< 0.001
	**7p**	WT<br>Gain	40<br>5	4<br>8	0.539	< 0.001
	**7q**	WT<br>Gain	39<br>4	6<br>8	0.503	0.001
	**8p**	WT<br>Loss	34<br>9	3<br>11	0.504	< 0.001
	**9p**	WT<br>Loss	35<br>7	1<br>14	0.679	< 0.001
	**9q**	WT<br>Loss	33<br>7	3<br>14	0.607	< 0.001
	**10q**	WT<br>Loss	39<br>6	3<br>9	0.565	< 0.001
	**14q**	WT<br>Loss	28<br>4	6<br>19	0.641	< 0.001

1 – Cohen's Kappa Test
