# Supplementary material for: DNA methylation associates with survival in non-metastatic clear cell renal cell carcinoma
Source: BMC Cancer. 2019 Jan 14;19:65. doi: 10.1186/s12885-019-5291-3 (PMC6332661; doi:10.1186/s12885-019-5291-3)
Supplement: Supplementary file 7 — Table S8. Top 20 most significant GO Terms for GeneIDs either hyper- or hypomethylated in M0-P and M1 tumor samples. (PDF 54 kb) [file 12885_2019_5291_MOESM7_ESM.pdf]

Additional Table 8.

Geneset Hypermethylated	Description	P-value	GeneIDs
GO:0050954	sensory perception of mechanical stimulus	0.000213929	40;491;3784;5077;9311;89797
GO:0010991	negative regulation of SMAD protein complex assembly	0.000335484	4091;57057
GO:0021563	glossopharyngeal nerve development	0.000335484	3213;89797
GO:0050915	sensory perception of sour taste	0.000335484	40;9311
GO:0050974	detection of mechanical stimulus involved in sensory perception	0.000359707	40;491;9311
GO:0010990	regulation of SMAD protein complex assembly	0.000501309	4091;57057
GO:1903025	regulation of RNA polymerase II regulatory region sequence-specific DNA binding	0.000699158	1649;64324
GO:0044058	regulation of digestive system process	0.000933445	2693;3784;4986
GO:0007605	sensory perception of sound	0.001061258	40;491;3784;5077;89797
GO:0015871	choline transport	0.001189446	6397;80736
GO:0061061	muscle structure development	0.001256607	1649;5077;7134;8470;30812;57057;84466;140578;150921;284358
GO:0009268	response to pH	0.001524463	40;3373;9311
GO:0050982	detection of mechanical stimulus	0.001763248	40;491;9311
GO:0001976	neurological system process involved in regulation of systemic arterial blood pressure	0.001803409	40;89797
GO:0003177	pulmonary valve development	0.002155863	4091;57057
GO:0003184	pulmonary valve morphogenesis	0.002155863	4091;57057
GO:0007183	SMAD protein complex assembly	0.002949933	4091;57057
GO:0007517	muscle organ development	0.003319831	5077;7134;30812;57057;84466;140578;150921
GO:2000251	positive regulation of actin cytoskeleton reorganization	0.003390845	2242;3055
GO:0072523	purine-containing compound catabolic process	0.003684015	5143;50940;51816
**Geneset Hypomethylated**	**Description**	**P-value**	**GeneIDs**
GO:0009605	response to external stimulus	1.38E-05	301;302;375;841;1235;1437;2865;3428;3553;4353;4600;5337;5723;6590;9021;90678
GO:0006952	defense response	2.72E-05	301;375;841;1235;2865;3428;3553;4261;4353;4600;6590;9021;90678
GO:0006955	immune response	3.33E-05	301;841;1235;1437;2865;3428;3553;4261;4600;6590;8530;9021;9404
GO:1901700	response to oxygen-containing compound	5.24E-05	301;535;841;1437;2788;2865;3553;4353;5723;6590;9021;23175;23209
GO:0010638	positive regulation of organelle organization	5.93E-05	301;302;375;841;908;1437;3553;90678
GO:0043434	response to peptide hormone	6.53E-05	301;535;2788;2865;3553;9021;23175
GO:0051707	response to other organism	9.31E-05	375;841;1437;3428;3553;4353;4600;6590;90678
GO:0043207	response to external biotic stimulus	9.41E-05	375;841;1437;3428;3553;4353;4600;6590;90678
GO:0071375	cellular response to peptide hormone stimulus	0.000107867	535;2788;2865;3553;9021;23175
GO:1901701	cellular response to oxygen-containing compound	0.000110827	301;535;1437;2788;2865;3553;4353;9021;23175;23209
GO:1901652	response to peptide	0.000119627	301;535;2788;2865;3553;9021;23175
GO:0032869	cellular response to insulin stimulus	0.000130264	535;2865;3553;9021;23175
GO:0009607	response to biotic stimulus	0.000138233	375;841;1437;3428;3553;4353;4600;6590;90678
GO:1901653	cellular response to peptide	0.000154636	535;2788;2865;3553;9021;23175
GO:0031340	positive regulation of vesicle fusion	0.000154844	301;302
GO:0043086	negative regulation of catalytic activity	0.000176425	301;302;841;3428;3553;6590;8530;9021;200734
GO:0031347	regulation of defense response	0.000205757	301;375;841;2865;3428;3553;9021;90678
GO:0002252	immune effector process	0.000208099	301;375;1235;2865;3428;3553;4353;4600
GO:0080134	regulation of response to stress	0.00029618	301;302;375;841;2865;3428;3553;9021;23209;90678;200734
GO:0033993	response to lipid	0.000315059	301;841;1437;2865;3553;4353;5723;6590;23209
