# Supplementary material for: DNA methylation associates with survival in non-metastatic clear cell renal cell carcinoma
Source: BMC Cancer. 2019 Jan 14;19:65. doi: 10.1186/s12885-019-5291-3 (PMC6332661; doi:10.1186/s12885-019-5291-3)
Supplement: Supplementary file 8 — Table S4. Cox’s proportional hazard regression analysis for cancer specific survival (CSS) in 115 ccRCC samples. (PDF 108 kb) [file 12885_2019_5291_MOESM8_ESM.pdf]

Additional Table 4

**Variables**		**Hazard Ratio**	**Hazard Ratio (95 % CI)**	***p-value***
Age		0.864	0.955 – 1.017	*0.353*
Gender	Female		Ref.	
	Male	2.220	1.087 – 4.533	*0.029*
TNM stage	I		Ref.	
	II	1.512	0.292 – 7.816	*0.622*
	III	6.921	2.284 – 20.970	*0.001*
	IV	33.868	11.158 – 102.805	< *0.001*
Morphological grade	G1 + G2		Ref.	
	G3 + G4	4.014	1.823 – 8.840	*0.001*
Cluster Status	A		Ref.	
	B	1.009	0.474 – 2.072	*0.982*
