# Supplementary material for: DNA methylation associates with survival in non-metastatic clear cell renal cell carcinoma
Source: BMC Cancer. 2019 Jan 14;19:65. doi: 10.1186/s12885-019-5291-3 (PMC6332661; doi:10.1186/s12885-019-5291-3)
Supplement: Supplementary file 9 — Table S5. Cox’s proportional hazard regression analysis for progress free survival (PFS) in 87 M0 ccRCC samples. (PDF 108 kb) [file 12885_2019_5291_MOESM9_ESM.pdf]

Additional Table 5

Variables		Hazard Ratio	Hazard Ratio (95 % CI)	*p-value*
Age		0.970	0.929 – 1.013	*0.165*
Gender	Female		Ref.	*0.212*
	Male	0.212	0.722 – 4.319	
TNM stage	I		Ref.	
	II	1.801	0.458 – 7.079	*0.399*
	III	4.985	1.579 – 15.738	*0.006*
Morphological grade	G1		Ref.	
	G2	1.634	0.337 – 7.928	*0.542*
	G3	3.427	0.553 – 21.227	*0.185*
	G4	7.139	1.047 – 48.653	*0.045*
Cluster Status	A		Ref.	*0.610*
	B	0.610	0.210 – 2.498	
