# Supplementary material for: DNA methylation associates with survival in non-metastatic clear cell renal cell carcinoma
Source: BMC Cancer. 2019 Jan 14;19:65. doi: 10.1186/s12885-019-5291-3 (PMC6332661; doi:10.1186/s12885-019-5291-3)
Supplement: Supplementary file 12 — Table S6. Clinicopathological parameters for tumors included in the heterogeneity analysis. (PDF 65 kb) [file 12885_2019_5291_MOESM12_ESM.pdf]

Additional Table 6

Patient	Diameter (mm)	Grade	T-stage	M-stage	TNM
1	43	2	T1b	M0	I
2	50	3	T1b	M0	I
4	55	2	T1b	M0	I
5	50	2	T1b	M0	I
6	85	2	T2	M0	II
