# Supplementary material for: DNA methylation associates with survival in non-metastatic clear cell renal cell carcinoma
Source: BMC Cancer. 2019 Jan 14;19:65. doi: 10.1186/s12885-019-5291-3 (PMC6332661; doi:10.1186/s12885-019-5291-3)
Supplement: Supplementary file 13 — Table S7. Cox’s proportional hazard regression analysis for progress free survival (PFS) in 87 M0 ccRCC samples. (PDF 108 kb) [file 12885_2019_5291_MOESM13_ESM.pdf]

Additional Table 7.

Variables		Hazard Ratio	Hazard Ratio (95 % CI)	*p-value*
Age		0.956	0.914 – 1.001	*0.056*
Gender	Female		Ref.	*0.181*
	Male	1.621	0.676 – 3.884	
TNM stage	I		Ref.	
	II	1.893	0.472 – 7.602	*0.368*
	III	3.890	1.376 – 10.998	*0.010*
Morphological grade	G1		Ref.	
	G2	0.950	0.179 – 5.026	*0.952*
	G3	1.967	0.337 – 11.474	*0.452*
	G4	5.314	0.826 – 34.194	*0.079*
PMC	Low		Ref.	*0.019*
	High	4.409	1.281 – 15.819	
